# Prediction of acupuncture efficacy in acute ischemic stroke constructing a clinical-radiomics multimodal model

**DOI:** 10.3389/fneur.2025.1682842

**Published:** 2025-11-28

**Authors:** Lijuan Zhao, Ziguang Huang, Baoying Zhao, Ran An, Yanyan Cheng

**Affiliations:** 1 The First Clinical College, Liaoning University of Traditional Chinese Medicine, Shenyang, China; 2 The Affiliated Hospital of Liaoning University of Traditional Chinese Medicine, Shenyang, Liaoning, China

**Keywords:** acute ischemic stroke, prediction model, acupuncture, radiomics, MRI

## Abstract

**Objectives:**

The aim of this study is to construct and validate a prediction model fusing multimodal radiomics and clinical features to evaluate the prognosis of acute ischemic stroke patients treated with acupuncture.

**Methods:**

This study retrospectively included 186 patients with acute ischemic stroke who received acupuncture treatment after stroke. The results of Barthel Index scores before and after treatment were used to determine whether acupuncture was effective or ineffective. All patients were randomly assigned to the training dataset (*n* = 126) or testing dataset (*n* = 60) on a 7:3 basis. First, collect baseline clinical data and pre-treatment radiomics data of the infarct lesions, subsequently, perform min-max normalization, then screen variables through Pearson correlation analysis and LASSO regression. Constructing clinical models, radiomics models, and combined models, and comparing the performance of Logistic Regression (LR), LightBoost, and K-Nearest Neighbors (KNN) algorithms in each model. Finally, the best model is selected based on the results of the testing dataset.

**Results:**

Four clinical features and eight radiomics features were finally screened to construct the model. Testing dataset results showed limited performance of the clinical model (AUC = 0.689–0.703) versus the radiomics model (AUC = 0.729–0.759), the combined model performed significantly better (KNN algorithm: AUC = 0.889) and its combined discriminative efficacy was outstanding (accuracy = 0.800, sensitivity = 0.914, specificity = 0.640, precision = 0.781).

**Conclusion:**

The combined model integrating clinical and radiomics features can accurately screen the population benefiting from acupuncture treatment, in which the KNN algorithm has the best stability and provides a reliable basis for individualized treatment decisions.

## Introduction

Acute ischemic stroke is one of the leading causes of death and disability globally (1), and its high incidence and disability pose a major challenge to public health systems (2). Epidemiological studies have shown significant regional differences in the distribution of ischemic stroke incidence: compared with the declining trend in most developed countries, the incidence in East Asia (especially China) continues to increase (3–4). In 2021, the total number of stroke patients in China reached 26 million, an increase of 104.26% compared with 1990, seriously affecting patients’ quality of life and life expectancy. As China’s population aging process accelerates, the stroke burden is expected to further intensify (5). Acupuncture, as a commonly used intervention in the rehabilitation phase of acute ischemic stroke, has the advantages of simplicity and fewer adverse effects. Early research primarily focused on validating its clinical efficacy for core symptoms such as neurological deficits and motor impairments in stroke patients (6–7). With the deepening of research, its mechanisms of action have gradually been revealed. Studies indicate that acupuncture can significantly improve clinical outcomes through various pathways, including reducing the inflammatory response, alleviating cerebral edema, and promoting neural repair (8–9). Current research in this field is further deepening through the integration of multidisciplinary approaches such as neuroimaging and molecular biology, aiming to reveal the intrinsic principles and network regulation mechanisms of acupuncture’s effects at a deeper level (10). Nevertheless, the practical application of acupuncture in the clinical rehabilitation of AIS still mainly relies on standardized treatment protocols and clinical experience, and there is a lack of biomarkers that can early and objectively predict individual patient therapeutic responses. Therefore, the development of accurate and effective tools for the early identification of predicting the functional recovery and prognostic effects of acupuncture in acute ischemic stroke patients is important for the development of personalized treatment plans and optimization of healthcare resource allocation.

Currently, prognostic prediction models for AIS based on conventional clinical indicators (e.g., age, medical history, laboratory findings) have been reported (11). Although they provide some guidance for clinical practice, these models have limitations in revealing lesion-specific pathophysiological details. Radiomics is an emerging medical image analysis technology that allows high-throughput extraction of a large number of quantitative features that are indistinguishable to the naked eye from computed tomography, magnetic resonance imaging (MRI) (12). These features are believed to reflect more objectively and comprehensively the microstructure, functional status and potential pathophysiological changes of tissues (13–14). With this powerful information mining capability, radiomics has demonstrated good predictive value in the diagnosis, grading and efficacy prediction of tumors (15–16), prognostic assessment of stroke (17–18) and risk stratification and characterization of cardiovascular diseases (19).

To this end, this study constructed a prediction model for predicting patients with acute ischemic stroke treated with acupuncture based on MRI radiomic features and clinical data. The Barthel Index scores, which is widely recognized internationally, was used to assess the patients’ ability to perform activities of daily living, and the change in pre- and post-treatment values was used as the primary efficacy endpoint to quantify the degree of functional improvement after acupuncture intervention. Three machine learning models, Logistic Regression (LR), K-Nearest Neighbors (KNN) and LightBoost, were constructed and compared to systematically evaluate the accuracy, robustness, and clinical application value of the multimodal models in predicting acupuncture efficacy, with the aim of identifying key predictors and selecting the optimal model. Unlike most studies (20–21) that focus on overall functional outcomes such as 90-day mRS, which reflect the natural course and comprehensive treatment effects, this study uses the change in BI before and after acupuncture as an intervention-related efficacy indicator, enabling the model not only to have prognostic function but also therapeutic diagnostic characteristics. This research model represents a critical step toward advancing from standardized treatment to personalized rehabilitation medicine, and is expected to provide an objective tool for early identification of populations that would best respond to acupuncture, thereby promoting more precise and individualized AIS rehabilitation treatment, optimizing healthcare resource allocation, and improving patient rehabilitation outcomes.

## Materials and methods

### Study population and inclusion/exclusion criteria

This retrospective study collected clinical and imaging data from acute ischemic stroke patients hospitalized at Liaoning University of Traditional Chinese Medicine Hospital between January 2022 and December 2024, and a total of 186 cases were included. Inclusion criteria: (1) Age ≥18 years; (2) Diagnosis of new-onset cerebral infarction confirmed by standard head MRI and DWI sequences before treatment; (3) Receipt of acupuncture treatment during hospitalization; (4) Availability of Barthel Index scores in admission and discharge medical records; (5) Absence of acupuncture contraindications (e.g., coagulation disorders, localized skin infections, severe cardiovascular/cerebrovascular disease, psychiatric history, motion sickness history, pregnancy or lactation). Exclusion criteria: (1) history of traumatic brain injury and surgery, acute cerebral hemorrhage; (2) brain tumor; (3) patients treated with stent implantation for cerebrovascular disease; (4) severe cardiac, hepatic, renal dysfunction and autoimmune diseases; (5) poor quality of MRI images.

### Ethical considerations

This study was approved by the Ethics Review Committee of the Affiliated Hospital of Liaoning University of Traditional Chinese Medicine (Approval No.: Y202520CS(KT) 208-01). Verbal informed consent was obtained from all participants or their legal representatives via telephone interviews, during which the data processing and privacy protection protocols were communicated. To protect participant confidentiality, all personally identifiable information was removed, resulting in a fully anonymized dataset safeguarded by encryption and strict access controls.

### Acupuncture treatment protocol

All patients received conventional body acupuncture treatment. Patients were placed in the supine position. The selected acupoints included: Hegu (LI4), Neiguan (PC6), Chize (LU5), Quchi (LI11), Yanglingquan (GB34), Yinlingquan (SP9), Fengshi (GB31), Zusanli (ST36), Sanyinjiao (SP6), and Taichong (LV3). Prior to each acupuncture session, the skin was thoroughly disinfected with alcohol. Fine needles of 0.25 × 40 mm specification (Huatuo brand disposable sterile acupuncture needles, Suzhou Medical Supplies Factory, China) were used and inserted vertically into the skin. Lifting, thrusting, twisting, and rotating manipulation techniques were applied to achieve the deqi sensation. After all needles were accurately inserted into the acupoints, they were retained for 30 min. The treatment was administered once daily.

### Efficacy assessment and grouping

This study employed the Barthel Index (BI) as the assessment tool for activities of daily living. The BI is a widely used functional independence scale in stroke rehabilitation research, demonstrating good reliability and validity (22–23). Its items cover 10 basic activities of daily living, including feeding, transfer, grooming, toileting, bathing, walking, ascending and descending stairs, dressing, bowel control, and bladder control, comprehensively reflecting changes in patients’ self-care ability. We used the change in the Barthel Index (ΔBI) as the efficacy evaluation indicator, where ΔBI = BI score at discharge − BI score at admission. Patients were divided into two groups based on ΔBI: the responsive group (ΔBI > 0, indicating functional improvement) and the non-responsive group (ΔBI ≤ 0, indicating no improvement or worsening of function). This grouping method is based on the sensitivity of the BI to functional changes and can intuitively reflect functional outcomes after rehabilitation intervention (24). All BI assessments were performed by uniformly trained rehabilitation physicians or therapists, with assessment time points within 24 h after patient admission and within 24 h before discharge. To ensure assessment consistency, we referred to the standardized Barthel Index assessment process recommended in the study by Duffy et al. (24) and conducted consistency training for assessors to reduce inter-rater variability.

### Clinical data collection

Baseline data were retrospectively collected on 186 eligible patient, including age, sex, hypertension, diabetes mellitus, coronary artery disease, dyslipidemia, stroke history, smoking history, history of alcohol consumption, white blood cell count, neutrophil count, monocyte count, lymphocyte count, platelet count, total cholesterol, triglyceride, HDL cholesterol, LDL cholesterol, and fasting blood glucose, multiple cerebral infarcts, other rehabilitation treatments, and Barthel Index scores before and after treatment.

### Image data collection

All MRI-DWI images (b-value = 1,000 s/mm^2^) were acquired using a Philips 3.0 T MRI scanner. The patient is placed in the standard supine position with the arms at the side of the body. Cranial DWI was performed using cross-sectional scanning, with the localization line parallel to the anterior–posterior angle of the corpus callosum in the median sagittal image; the position and angle were adjusted in the coronal and cross-sectional planes to make the image centered and symmetrical; T1WI/T2WI cross-sectional and T1WI sagittal scans were performed, with the parameter settings of layer thickness of 6 mm, layer spacing of 1 mm, FOV of 232 × 125 mm, matrix of 108 × 130, and T2WI Sequence TR/TE = 2,476/97 ms. Based on the above images, manual segmentation of acute cerebral infarction lesions was performed independently by an attending radiologist with 5 years of experience, outlining regions of interest along the edges of the infarcted areas. For multiple cerebral infarction cases, critically outlines all imaging-visible acute infarct lesions (including small scattered lesions) to ensure maximum coverage of potentially predictive relevant lesions. Once the images were outlined, they were reviewed and confirmed by another senior imaging physician.

### Feature selection and model construction

Reviewed ROI images anonymized and imported into 3D-Slicer software in DICOM format (version 4.10.2). Based on the finalized ROIs, seven classes of radiomics features were extracted by 3D-Slicer’s Radiomics plug-in package. It contains a first-order histogram (Firstorder), gray-level covariance matrix (GLCM), gray-level dependency matrix (DIDM), gray-level size region matrix (GLSZM), gray-level tour matrix (GLRLM), neighborhood gray-level difference matrix (NGTDM), and morphological features (shape), totaling 851 features.

The Neusoft Explore Multimodal Medical AI Platform was used to construct the prediction model, and 186 patients were randomly divided into the training dataset and the test dataset in the ratio of 7:3. The training dataset was used for model construction and the testing dataset was used to validate the predictive efficacy of the model for the clinical efficacy of acute ischemic stroke acupuncture rehabilitation therapy. In the training dataset, feature screening and modeling were performed on the collected clinical variables and radiomics features, respectively. The min-max normalization is first performed on all features, and the data are scaled to the range [0, 1] by a linear transformation, calculated as:


$$X_{\mathrm{new}}=\frac{X-X_{\min}}{X_{\max}-X_{\min.}}$$

**Figure 1:**
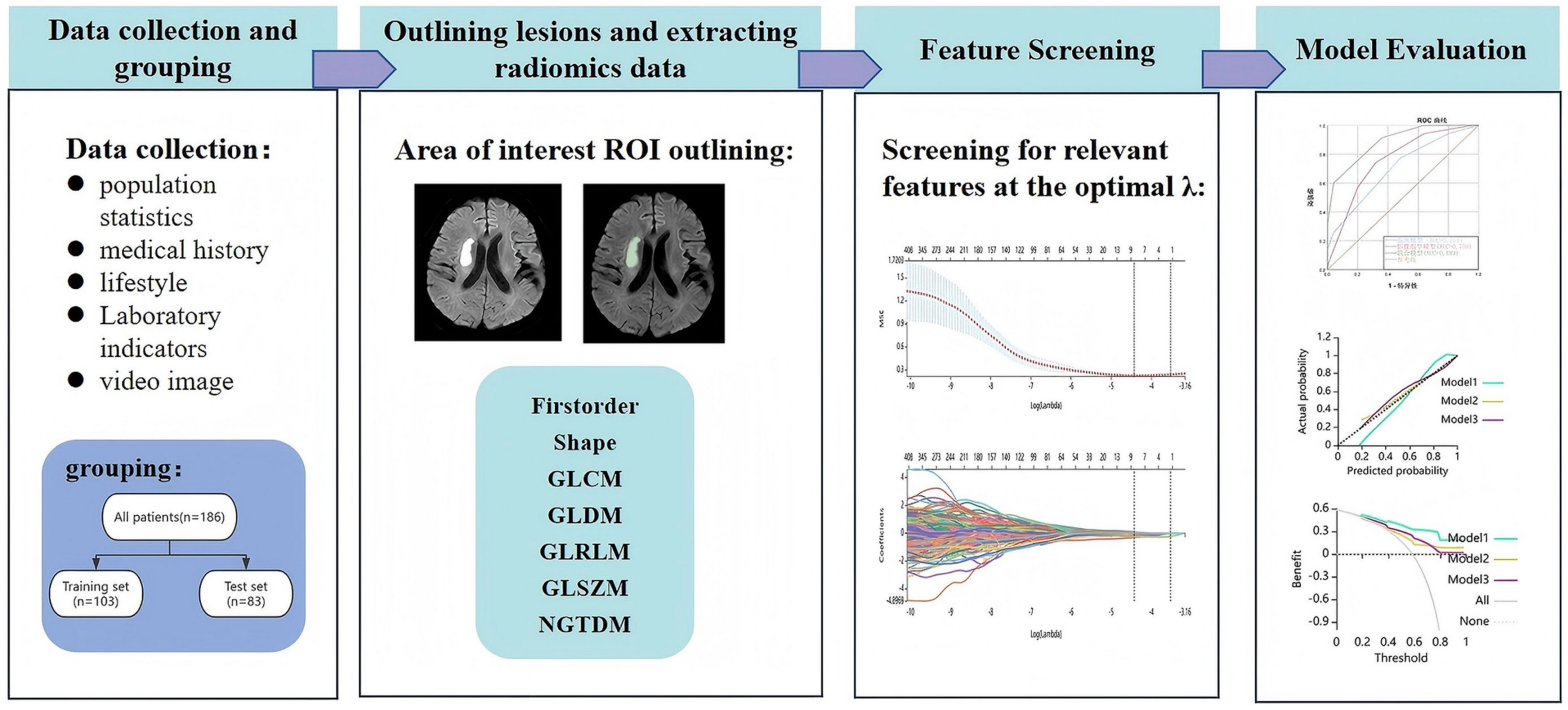
Experimental flowchart.

Where X denotes the radiomics characterization raw data, and X_min_ and X_max_ are the minimum and maximum values in the dataset, respectively. The initial selection of features was then analyzed by Pearson correlation analysis, and features were downscaled and selected based on LASSO regression and 10-fold cross-validation to identify predictors. Based on the finalized feature set, clinical scores, radiomics scores and combine scores are calculated separately. Then three machine learning algorithms, LR, KNN and LightBoost, were, respectively, employed to construct the clinical model, radiomics model and combined model. The specific process is shown in Figure 1.

### Statistical analysis

Data analysis was conducted using SPSS 26.0 software and Neusoft Explore Multimodal Medical Artificial Intelligence Platform. In order to analyze the variability of the baseline characteristics between the training and test dataset. An independent samples t-test was performed for values that conformed to a normal distribution, and values are expressed as mean ± standard deviation. Mann–Whitney U test for non-normally distributed measures, described by median (quartiles) [M (P25, P75)]. Count data were expressed as frequencies (percentages) using the chi-square test. ROC curves, calibration curves, and DCA curves were plotted to comprehensively assess model performance metrics such as sensitivity, specificity, accuracy, F1 scores, and area under the curve (AUC). DeLong test was used to compare different models AUC. Degree of compliance of calibration curve analysis with actual probabilities. DCA curve analysis clinical net benefits. Differences were considered statistically significant at *p* < 0.05.

## Results

### Patient baseline data situation

A total of 186 patients with acute ischemic stroke (103 in the effective group and 83 in the ineffective group) were enrolled in this study and randomized into a training dataset (*n* = 126) and a test dataset (*n* = 60) in a 7:3 ratio. As shown in Table 1, the difference in age and days of illness between the two groups in the training and testing datasets was statistically significant (*p* < 0.05). While gender, hypertension, diabetes mellitus, coronary artery disease, dyslipidemia, stroke history, smoking history, history of alcohol consumption, multiple infarct lesions, number of days of acupuncture treatment, white blood cell count, neutrophil count, lymphocyte count, monocyte count, platelet count, triglyceride, total cholesterol, low-density lipoprotein cholesterol, high density lipoprotein cholesterol, fasting blood glucose, pre-treatment Barthel Index score, and whether or not they had undergone rehabilitation training, there was no statistical difference (*p* > 0.05).

**Table 1:** Comparison of basic clinical characteristics of acute ischemic stroke patients in training dataset and test dataset.

Parameter	Training dataset (*n* = 126)			Testing dataset (*n* = 60)		
	Valid group (*n* = 68)	Invalid group (*n* = 58)	*p* value	Valid group (*n* = 35)	Invalid group (*n* = 25)	*p* value
Age, years	66.78 ± 10.13	70.47 ± 9.13	0.035	64.94 ± 9.56	71.04 ± 8.94	0.015
Sex (%)			0.542			0.753
Male	48 (38.10)	38 (30.16)		21 (35.00)	16 (26.67)	
Female	20 (15.87)	20 (15.87)		14 (23.33)	9 (15.00)	
Hypertension, *n* (%)			0.915			0.315
Yes	44 (34.92)	37 (29.37)		20 (33.33)	11 (18.33)	
No	24 (19.05)	21 (16.67)		15 (25.00)	14 (23.33)	
Diabetes, *n* (%)			0.422			0.961
Yes	35 (27.78)	34 (26.98)		25 (41.67)	18 (30.00)	
No	33 (26.19)	24 (19.05)		10 (16.67)	7 (11.67)	
Coronary heart disease, *n* (%)			0.132			0.128
Yes	4 (3.17)	8 (6.35)		8 (13.33)	2 (3.33)	
No	64 (50.79)	50 (39.68)		27 (45.00)	23 (38.33)	
Dyslipidemia (%)			0.669			0.778
Yes	15 (11.90)	11 (8.73)		6 (10.00)	5 (8.33)	
No	53 (42.06)	47 (37.30)		29 (48.33)	20 (33.33)	
Previous stroke, *n* (%)			0.614			0.445
Yes	24 (19.05)	23 (18.25)		12 (20.00)	11 (18.33)	
No	44 (34.92)	35 (27.78)		23 (38.33)	14 (23.33)	
Smoking (%)			0.723			0.513
Yes	18 (14.29)	17 (13.49)		8 (13.33)	4 (6.67)	
No	50 (39.68)	41 (32.54)		27 (45.00)	21 (35.00)	
Alcohol consumption (%)			0.733			0.937
Yes	8 (6.35)	8 (6.35)		3 (5.00)	2 (3.33)	
No	60 (47.62)	50 (39.68)		32 (53.33)	23 (38.33)	
Multiple infarct lesions (%)			0.909			0.895
Yes	38 (30.16)	33 (26.19)		16 (26.67)	11 (18.33)	
No	30 (23.81)	25 (19.84)		19 (31.67)	14 (23.33)	
Days of illness	1 (1, 2)	2 (1, 4.25)	0.034	1 (1, 3)	1 (1, 3)	0.087
Acupuncture days	13 (10.25, 13.75)	12 (10, 13)	0.327	12 (11, 13)	13 (11, 13.50)	0.359
White blood cell count, 10^9^/L	6.86 ± 1.94	6.80 ± 2.16	0.878	7.35 (5.82, 7.88)	5.91 (5.60, 7.93)	0.301
Neutrophil count, 10^9^/L	4.62 ± 1.70	4.58 ± 1.84	0.886	4.72 (3.70, 5.13)	3.90 (3.58, 4.92)	0.254
Lymphocyte count, 10^9^/L	1.74 ± 0.58	1.69 ± 0.60	0.628	1.86 ± 0.69	1.71 ± 0.59	0.364
Monocyte count, 10^9^/L	0.41 ± 0.12	0.38 ± 0.12	0.145	0.37 ± 0.88	0.41 ± 0.12	0.198
Platelet count, 10^9^/L	217.91 ± 52.23	210.62 ± 53.75	0.442	216.94 ± 56.29	220.52 ± 52.75	0.804
Triglyceride, mmol/L	1.37 (0.97, 1.98)	1.61 (1.07, 2.11)	0.195	1.26 (1.03, 1.58)	1.51 (1.17, 1.91)	0.197
Total cholesterol, mmol/L	4.65 (3.96, 5.50)	4.46 (4.09, 5.03)	0.191	4.49 ± 1.20	4.69 ± 0.85	0.483
HDL cholesterol, mmol/L	1.15 (0.97, 1.44)	1.18 (0.93, 1.57)	0.644	1.02 (0.82, 1.33)	1.08 (0.86, 1.52)	0.440
LDL cholesterol, mmol/L	2.69 ± 0.94	2.35 ± 1.01	0.051	2.36 ± 0.91	2.72 ± 0.84	0.125
Fasting blood glucose, mmol/L	7.45 (5.90, 9.15)	7.20 (6.1, 9.1)	0.979	8.10 (6.60, 9.10)	7.40 (6.25, 8.55)	0.329
Pre-treatment Barthel score	72.50 (40, 90)	65 (43.75, 85)	0.493	60 (40, 85)	80 (52.50, 90)	0.161
Rehabilitation (%)			0.794			0.880
Yes	79 (42.47)	65 (34.94)		26 (43.33)	19 (31.667)	
No	24 (12.9)	18 (9.67)		9 (15.00)	6 (10.00)	

### Feature screening results

After the above process, the clinical model at the optimal *λ* value (*λ* = −4.603706220608013) finally screened and included four key clinical characteristics: age, days of illness, stroke history, and hypertension. They respectively denote the patient’s underlying physiologic status, time window of therapeutic intervention, previous cerebrovascular events, and vascular status. The radiomics model, on the other hand, screened eight most closely correlated group features at the optimal *λ* value (*λ* = −4.4826998342853885) from the initial 851 extracted features, one morphological feature (shape) with seven textural features (including GLRLM/GLDM/GLSZM/NGTDM), as shown in Table 2.

**Table 2:** Eight features screened based on radiomics features by LASSO dimensionality reduction.

Serial number	Image type	Characteristic category	Characterization
1	Original	shape	MajorAxisLength
2	Original	glrlm	RunLengthNonUniformity
3	Wavelet-HLL	gldm	LowGrayLevelEmphasis
4	Wavelet-HLL	glrlm	HighGrayLevelRunEmphasis
5	Wavelet-HLL	glszm	LowGrayLevelZoneEmphasis
6	Wavelet-HLL	glszm	ZoneEntropy
7	Wavelet-LLH	ngtdm	Strength
8	Wavelet-LLL	glrlm	RunLengthNonUniformity

**Figure 2:**
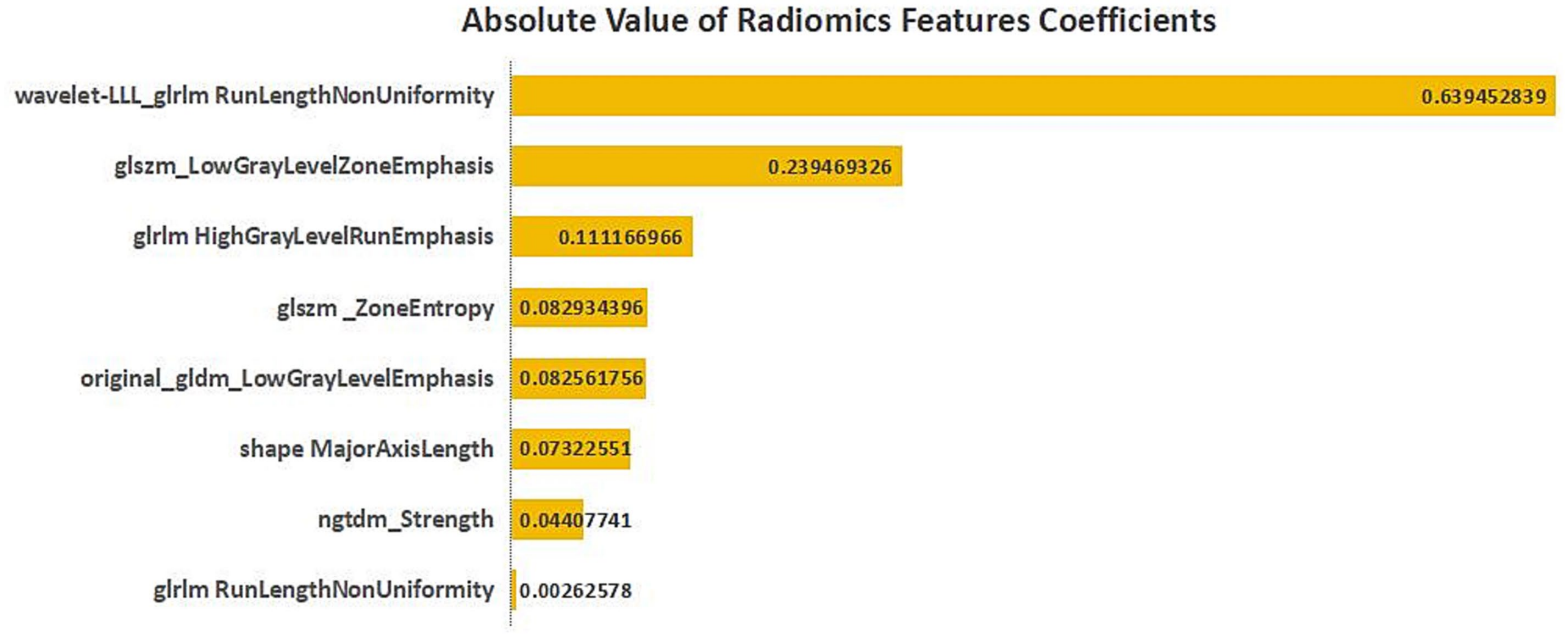
Feature importance ranking of the eight selected radiomics features. The ranking was generated based on the absolute values of the coefficients in the LASSO regression model. Features are displayed in descending order of their absolute coefficients, which reflect their relative contribution to the predictive model.

Based on the absolute values of the coefficients of each feature in the LASSO regression model, we ranked the importance of the selected eight radiomics features, and the results are shown in Figure 2. This ranking reflects the relative importance of different features to the predictive model.

These features reflect the pathology of acute cerebral infarct lesions at multiple levels by quantifying spatial extensibility, microscopic heterogeneity, and high- and low-gray-scale pathologic compartmentalization of the foci, and provide a basis for explaining the imaging mechanisms underlying the differences in acupuncture efficacy. Based on the key features screened above, we calculated the clinical score and the radiomics score, respectively. The score is a regression value calculated based on the LASSO regression model. The general formula for this score is:


$$\text{Regression Value}(\text{Score})=\beta_{0}+\beta_{1}f_{1}+\beta_{2}f_{2}+\ldots \ldots +\beta_{n}f_{n}$$

Among them, β_0_ is the model intercept (constant term), β_n_ is the regression coefficient of the n-th feature, and f_n_ is the value of the n-th feature. Each feature value f_n_ is multiplied by its corresponding coefficient β_i_, and the results of all products are summed. Then, the constant term β_0_ is added to obtain the final score. Therefore, the formulas for the clinical score and the radiomics score are as follows:


$$\begin{array}{c} \text{the clinical score}=0.015149013666327588-0.3432321932874825\\ \times \text{hypertension}-0.2806378057487655\\ \times \text{days of illness}-0.0020073460881445348\\ \times \mathrm{age}+0.7742321909682528\times \text{stroke history} \end{array}$$

$$\begin{array}{c} \;\text{the radiomics score}=-0.25194588690119596\\ +0.07322551023505326\\ \times \text{orginal}\_\text{shape}\_\text{MajorAxisLength}\\ -0.08256175645106456\times \text{wavelet}\\ -\mathrm{HLL}\_\text{gldm}\_\text{LowGrayLevelEmphasis}\\ +0.11116696555522333\times \text{wavelet}\\ -\mathrm{HLL}\_\text{glrlm}\_\text{HighGrayLevelRunEmphasis}\\ -0.2394693262138793\times \text{wavelet}\\ -\mathrm{HLL}\_\text{glszm}\_\text{LowGrayLevelZoneEmphasis}\\ +0.08293439626993877\times \text{wavelet}\\ -\mathrm{HLL}\_\text{glszm}\_\text{ZoneEntropy}\\ -0.04407740973427451\times \text{wavelet}\\ -\mathrm{LLH}\_\text{ngtdm}\_\text{Strength}\\ +-0.002625779929787255\\ \times \text{original}\_\text{glrlm}\_\text{RunLengthNonUniformity}\\ +0.6394528393922362\times \text{wavelet}\\ -\mathrm{LLL}\_\text{glrlm}\_\text{RunLengthNonUniformity} \end{array}$$

### Model performance comparison

**Figure 3:**
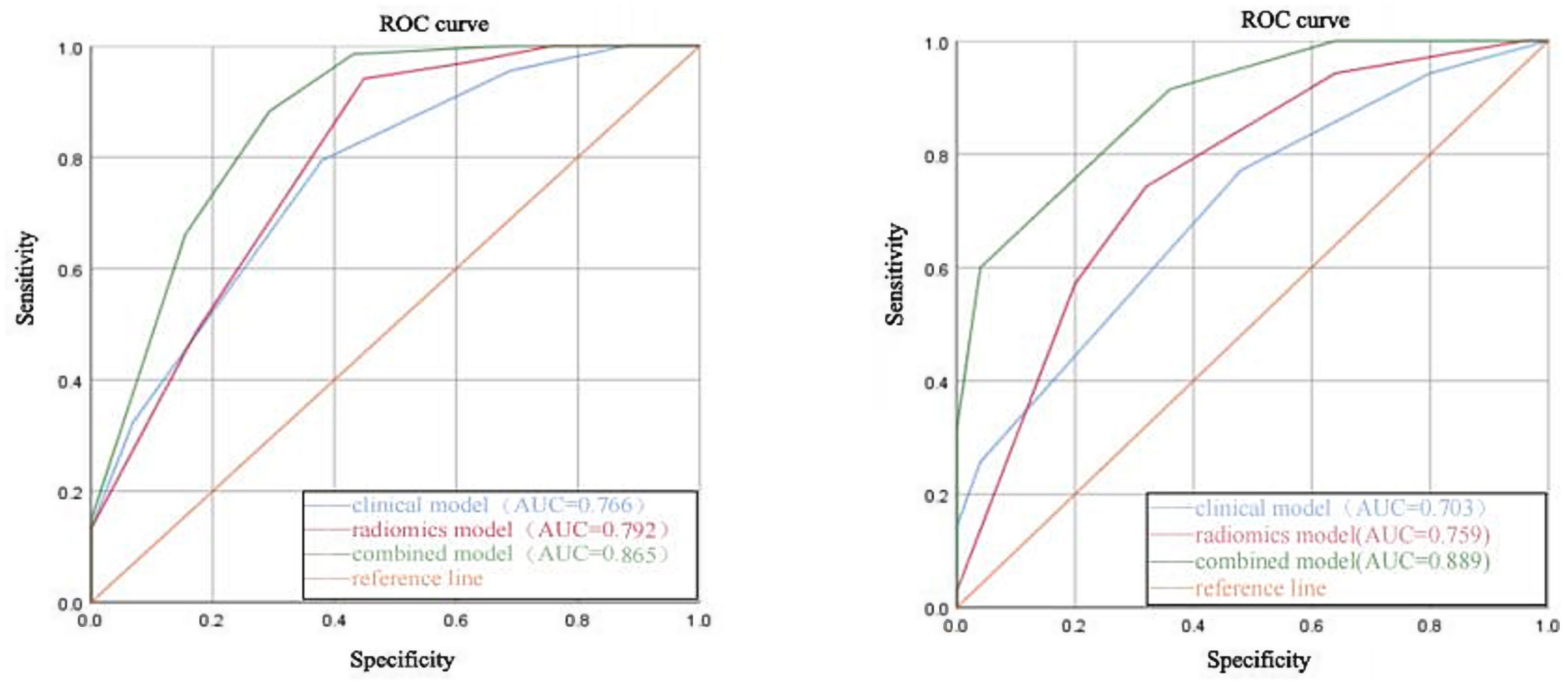
The left graph shows the ROC curves and AUC values of the KNN algorithm for the three models in the training dataset, and the right graph shows the ROC curves and AUC values of the KNN algorithm for the three models in the test dataset.

The results of the test dataset of all three models were optimal for the KNN algorithm, with the radiomics model AUC = 0.759 [95% CI: 0.633–0.886], which was superior to the clinical model AUC = 0.703 [95% CI: 0.571–0.834], and the combined model AUC was the highest at AUC = 0.889 [95% CI: 0.809–0.968]. DeLong’s test showed that there was no significant difference in AUC between the training and testing dataset (*p* > 0.05). Figure 3 shows the comparison of the ROC curves and AUC performance of the three models based on the KNN algorithm in the training dataset and the testing dataset.

**Figure 4:**
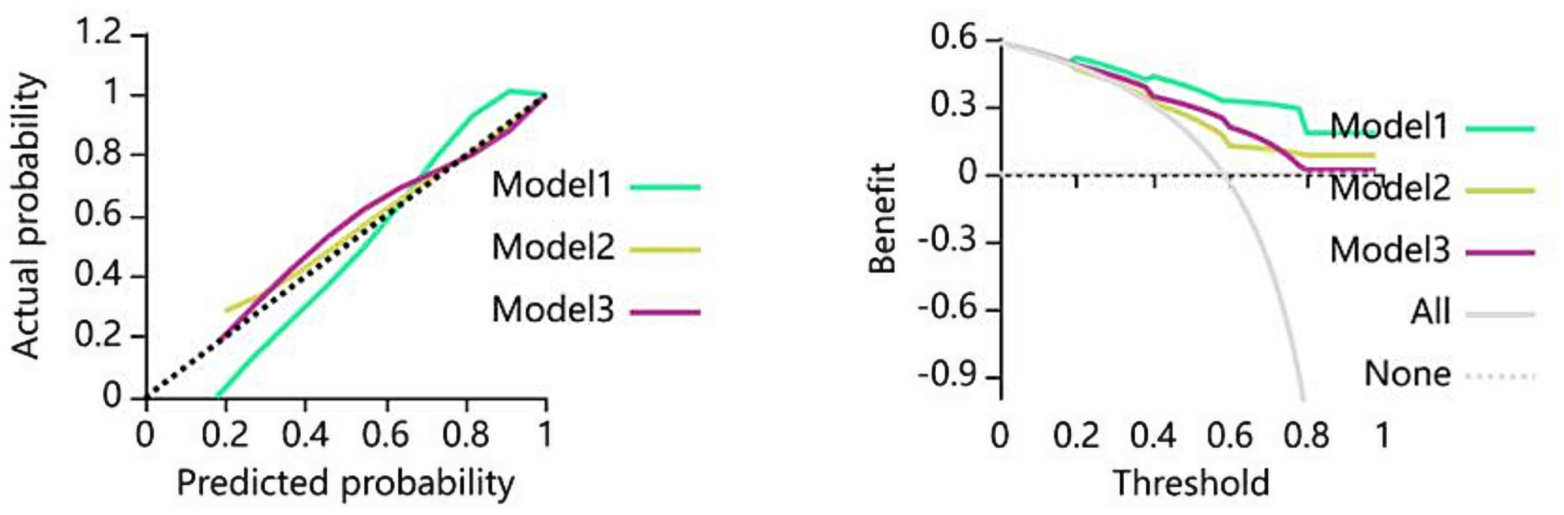
Calibration (Left) curves and decision curve analysis (Right) of three models using the KNN algorithm in the test cohort. The left panel displays calibration curves for the clinical model (Model 1), radiomics model (Model 2), and combined model (Model 3). Closer alignment between calibration curves and the reference diagonal (dashed line) indicates superior calibration performance. The right panel shows decision curves where the x-axis represents threshold probability and the y-axis denotes net benefit. The combined model (red) demonstrates the highest net benefit across clinically relevant thresholds.

The calibration curves of the clinical model, the radiomics model, and the combined model test dataset based on the KNN algorithm are shown in Figure 4 (Hosmer-Lemeshow test *p* > 0.05), which validate the goodness of fit of each model and confirms the reliability of the prediction of acupuncture efficacy for patients with acute ischemic stroke. And the net clinical benefit was assessed by decision curve analysis (DCA), and Figure 4 shows that the combined model of the test dataset consistently outperformed the other models within a reasonable threshold interval, providing a higher net benefit.

The results of the performance evaluation of the three models based on the training dataset and test dataset under LR, LightBoost, and KNN algorithms are shown in Table 3.

**Table 3:** Training dataset-test dataset performance comparison of multiple models in clinical, radiomics, and combined modalities.

Model	Model	AUC (95%CI)		Accuracy		Sensitivity		Specificity		Precision		f1-score	
		Training	Test	Training	Test	Training	Test	Training	Test	Training	Test	Training	Test
Clinical model	LR	0.681 [0.589, 0.775]	0.689 [0.555, 0.825]	0.627	0.650	0.779	0.800	0.448	0.440	0.624	0.667	0.693	0.727
	LightBoost	0.795 [0.716, 0.875]	0.693 [0.558, 0.829]	0.746	0.667	0.838	0.771	0.638	0.520	0.731	0.692	0.781	0.730
	KNN	0.766 [0.685, 0.849]	0.703 [0.571, 0.834]	0.714	0.667	0.794	0.771	0.621	0.520	0.711	0.692	0.750	0.730
Radiomics model	LR	0.735 [0.646, 0.823]	0.729 [0.598, 0.860]	0.706	0.683	0.809	0.771	0.586	0.560	0.696	0.711	0.748	0.740
	LightBoost	0.809 [0.734, 0.885]	0.745 [0.617, 0.873]	0.762	0.700	0.897	0.714	0.603	0.680	0.726	0.758	0.803	0.735
	KNN	0.792 [0.713, 0.871]	0.759 [0.633, 0.886]	0.762	0.717	0.941	0.742	0.551	0.680	0.711	0.765	0.810	0.754
Combined model	LR	0.805 [0.728, 0.881]	0.793 [0.679, 0.907]	0.746	0.717	0.779	0.771	0.707	0.640	0.757	0.750	0.768	0.761
	LightBoost	0.907 [0.853, 0.961]	0.805 [0.694, 0.916]	0.833	0.700	0.882	0.857	0.776	0.480	0.823	0.698	0.851	0.769
	KNN	0.865 [0.801, 0.930]	0.889 [0.809, 0.968]	0.802	0.800	0.882	0.914	0.707	0.640	0.779	0.781	0.828	0.842

## Discussion

In this study, three prediction models were constructed based on radiomic features and multiple clinical factors, and the optimal model was screened by three machine learning algorithms for predicting the prognosis of acupuncture treatment in patients with acute ischemic stroke. It was found that the combined model (KNN machine algorithm) performed best in predicting the efficacy of acupuncture (test set AUC = 0.889), suggesting that combining the microscopic imaging characteristics of the lesion before treatment with the patient’s macroscopic clinical background can effectively reflect their potential responsiveness to acupuncture intervention.

The core innovation of this study lies in shifting the predictive focus from the general functional prognosis of stroke to the prediction of the response to a specific rehabilitation intervention (acupuncture). Unlike traditional prognostic models that predict comprehensive outcomes involving multiple treatments, the model constructed in this study is specifically designed to predict the patient’s likelihood of responding to acupuncture. This shift gives the model a “theranostic” attribute, and its clinical significance is that it can provide a direct basis for formulating individualized rehabilitation plans: for patients predicted by the model to be potential responders, acupuncture treatment can be actively adopted; while for those predicted to be non-responders, ineffective intervention can be avoided, and other rehabilitation strategies can be switched to or combined as early as possible.

In terms of model selection, the KNN algorithm showed significant advantages in this study. Its core advantage is that it does not make strong assumptions about the data distribution and can naturally capture complex patterns through local similarity (25), which highly matches the complex nonlinear relationships present in our clinical data. The KNN algorithm predicts by simply finding the ‘most similar’ neighbors. This mechanism enables it to naturally adapt to and capture this complex relationship that may not be easily expressed in simple mathematical form. For the clinical prediction problem targeted in this study, a simple model like KNN achieves the best balance between prediction performance and model complexity—a characteristic that is particularly beneficial for its final clinical translation application. Compared with more complex models such as LightBoost, KNN has relatively few hyperparameters and is easy to interpret (mainly the k value and distance metric). Although KNN does not provide coefficients like linear models, we can infer which features are most important in defining ‘similarity’ by examining samples that are frequently selected as neighbors. This provides a feasible path to understand the potential phenotypes or subgroups of the disease.

In recent years, several multi-center studies with rigorous methodology have made important contributions to the field of stroke prognosis prediction. Liu et al. (11) developed a deep learning model integrating DWI images and clinical features, which achieved AUCs of 0.92 and 0.90 for predicting poor functional outcomes in internal and external test sets, respectively; Jo et al. (26) used an automated machine learning framework to integrate multimodal features, and the AUC of their optimal combined model was 0.786; and a multi-center study based on MRI radiomics (27) showed that the clinical-radiomics combined logistic regression model had an AUC of 0.860. In this research context, the KNN combined model constructed in this study achieved an AUC value of 0.889. This performance is outstanding among current clinical-radiomics prediction models. This result further confirms the effectiveness of KNN as a simple model, indicating that while maintaining high predictive performance, it also has better potential for clinical application. In summary, the KNN algorithm has good potential in characterizing the complex feature-outcome relationship in stroke prognosis prediction, and the constructed model has reliable predictive performance.

Regarding clinical factors, this study employed various statistical methods including LASSO regression (with 10-fold cross-validation) and Pearson correlation analysis to rigorously screen patients’ clinical baseline data. It was found that age, history of hypertension, history of stroke, and days from onset to first acupuncture treatment were all independent risk factors affecting the efficacy of acupuncture in AIS. Research indicates that increasing age is often accompanied by decreased neuroplasticity and functional compensation capacity (28), which may be one reason for the attenuated response to acupuncture in elderly patients. Therefore, for this population, early intervention combining acupuncture with other rehabilitation techniques is recommended to enhance overall therapeutic efficacy. This study also found that AIS patients with comorbid hypertension demonstrated better responses to acupuncture. This phenomenon may be related to acupuncture’s role in adjunctively regulating blood pressure and improving cerebral blood flow perfusion (29). Systematic review studies have indicated that acupuncture can significantly reduce both systolic and diastolic blood pressure in hypertensive patients (30), providing supporting evidence for this explanation. The therapeutic effect of acupuncture relies on its regulatory function on the central nervous system (31). Consequently, for patients with a history of stroke, pre-existing neurological impairments may diminish this regulatory potential, becoming a key intrinsic factor affecting acupuncture efficacy (32). Therefore, clinical treatment should comprehensively consider the characteristics of previous brain damage, assess potential treatment risks and benefits, and formulate more refined acupuncture protocols. On the other hand, the days from onset to first acupuncture treatment reflects the importance of therapeutic timing during the acute phase. Early intervention can effectively improve AIS recovery (33), highlighting the clinical importance of administering acupuncture within the acute time window.

Radiomics can extract high-throughput features from medical images that exceed the recognition ability of the human eye, revealing potential heterogeneous information of the disease. This technology has been widely used in the evaluation of ischemic penumbra in stroke, prediction of neurological functional prognosis, and support of treatment decisions, providing an important non-invasive tool for precise diagnosis and treatment of stroke (34–36). Based on this, this study introduced radiomics features on the basis of traditional clinical indicators, aiming to extract objective information related to prognosis from the microscopic level of the lesion. Through strict screening, eight key features were finally determined, including one morphological feature and seven texture features. The morphological feature original_shape_MajorAxisLength directly reflects the infarct size, and the size of the infarcted area is a key factor affecting neurological recovery (37). The texture feature wavelet-HLL_gldm_LowGrayLevelEmphasis has high significance in identifying lesions (38). It represents the extent of distribution of low gray areas in the lesion, and the more extensive the distribution, the more severe the degree of tissue necrosis is likely to be. This feature was negatively correlated with the efficacy of acupuncture and may be related to the fact that extensive necrotic tissue indirectly attenuates the effect of acupuncture by causing irreversible nerve damage and loss of regenerative capacity (38). This finding directly links “lesion heterogeneity” with “therapeutic response,” and echoes the conclusion of Wei et al. that heterogeneity features (such as TotalEnergy) are independent predictors of long-term functional outcome (27). These evidences collectively indicate that highly heterogeneous infarct foci generally correspond to poorer neural recovery potential, and there may be a common pathological microenvironment behind it that is not conducive to repair. In addition, this study also analyzed the potential biological significance of other texture features. The abnormality of original_glrlm_RunLengthNonUniformity suggests that lesion heterogeneity not only originates from mixed pathological states such as cytotoxic edema and vasogenic edema, but may also reflect the voxel-level diffusion restriction differences caused by the asynchronous process of blood–brain barrier disruption at the microscopic level (27). The gray level size zone matrix features wavelet-HLL_glszm_ZoneEntropy and wavelet-HLL_glszm_LowGrayLevelEmphasis may reflect the spatial heterogeneity of the degree of tissue necrosis and the integrity information of the ischemic penumbra.

By linking these radiomics features to the neuroprotective mechanisms of acupuncture, we speculate that infarct lesions with high microstructural heterogeneity may represent a highly unstable or inflammation-active microenvironment. Such tissues may have a poor response capacity to the repair effects mediated by acupuncture, such as anti-inflammation, promotion of neuroplasticity, and improvement of cerebral perfusion. This speculation is also confirmed by a systematic evaluation, which shows that acupuncture can induce brain function activation in patients with ischemic stroke at the macroscopic level (39). Therefore, radiomics not only supplements the deficiency of clinical indicators from the microscopic level but also provides a biological explanation based on imaging for understanding the individual differences in acupuncture efficacy.

The combined model constructed in this study integrates clinical and radiomics features, and its performance is significantly better than that of a single model, indicating that the two have complementary effects in predicting acupuncture efficacy. Clinical indicators reflect the patient’s systemic status and basic disease background, while radiomics features quantify the local characteristics of the lesion itself. The combination of the two achieves a more comprehensive assessment of the prognosis. Nevertheless, its specificity (0.640) still needs to be improved, which means that at the current threshold, the model may have a certain proportion of false positive results, that is, misidentifying some healthy individuals or non-responders as positive. From the perspective of clinical practice, lower specificity may bring the risk of overtreatment. Therefore, optimizing the specificity of the model is crucial for its future clinical translation. It is worth noting that, in contrast, our combined model shows extremely high sensitivity (0.882), which is a more critical indicator in many clinical scenarios, especially for the prognosis prediction of AIS acupuncture treatment, because it can minimize missed diagnosis. In response to how to improve the specificity of the model, we plan to adopt the following strategies in future research: We will explore other integrated learning methods, such as gradient boosting trees (e.g., Extreme Gradient Boosting) or support vector machines (SVM). Such models usually perform better in processing complex feature interactions and improving the overall performance of the model, especially specificity. We will devote ourselves to finding and integrating new features that can better distinguish true positives from false positives. For example, we can consider adding the infarct location and National Institutes of Health Stroke Scale score, etc. These features are expected to provide complementary information, thereby effectively improving the specificity of the model. Afterwards, validate and recalibrate the model in a larger and more diverse independent cohort to reduce overfitting and improve generalization ability.

This study has some limitations: firstly, it is a single-center retrospective study with a small sample size and lack of external validation in an independent cohort, which limits the generalizability of the model results to some extent. Future sample size needs to be expanded and validated using multicenter prospective studies, and external validation of the model through independent cohorts across regions and populations to further enhance the universality and reliability of the research conclusions. Secondly, the lesion area is manually outlined, which may introduce subjective bias, and subsequent automatic segmentation methods based on deep learning can be explored to extract features to improve model accuracy.

## Conclusion

In summary, the combined prediction model constructed based on radiomics and clinical features is significantly better than the single model, providing incremental value for predicting the efficacy of acupuncture in AIS patients. This model links the microscopic radiomics features with the macroscopic efficacy of a specific therapy (acupuncture), which not only gives it the “theranostic” potential to guide individualized treatment but also opens up a new way to understand and verify the neurobiological mechanism of acupuncture from the imaging level.

## Data Availability

The raw data supporting the conclusions of this article will be made available by the authors without undue reservation.
